# Letter from Dr. A. Hill, Norwalk, Ct.

**Published:** 1848-10

**Authors:** 


### ARTICLE II.

### *Letter from* Dr. A. Hill, *Norwalk, Ct.*

Dr. Cone:—

Dear Sir:—I take pleasure in acknowledging your kindness in forwarding to me an extract from your report on "practical dentistry," containing your strictures on our new compound for filling carious teeth, known as "Hill's stoppings."

And although I am compelled to take a different view of the subject, as set forth in that extract, yet I appreciate the courtesy extended to me, in allowing me a copy prior to its being put to press.

The doctrine of the extract above referred to, after a careful analysis, may be correctly stated as follows: "Those who hold the highest stations in the profession, generally urge that any dental organ that can be preserved without injury to the surrounding parts, can be plugged with gold foil. Consequently, anything that claims to do more, must be injurious—anything less, unworthy of consideration. 'Hill's stopping' is mainly supported on the ground, that it can save teeth which cannot be saved with gold; therefore, it is to be inferred that 'Hill's stopping' is both injurious and unnecessary."

Believing this summary to contain the gist of your objections against our compound, I beg leave to submit the following remarks:

*First.*—If Dr. Cone, or any other member of the profession, will have the goodness to refer to, and carefully peruse our circular, setting forth the claims of this new compound, he or they will perceive that "the most available argument for its use," is not the simple fact that it can be used where gold cannot be. This is only *one* consideration among *many*, that enhances its claims to the consideration of the profession. But allowing for a moment, that this is the *only* argument that can be adduced in its favor, it does not by any means follow, that there are not other considerations in connection with it, capable of supporting its claims, even though it be placed in no tooth cavity where gold foil may not be packed. For, if it can be shown that it is less expensive, (and consequently much more extensive in its application,) and much easier to be put in any cavity, both as it regards the comfort of the patient and the labor of the dentist, then, we think, we have said something in its favor, worthy of consideration. And we aver both the one and the other.

The objections urged in the report are mainly hypothetical, and are not based upon actual experiment. The intelligent author *does not* say that the statements contained in the circular, that first heralded the discovery, *are not true;* nor does he give his readers to understand that he has made trial of the material, and failed to succeed. But it will be observed, that the report is based upon the presumption that gold is *all that is necessary*, and *all* that the "physiological and pathological dentist" can desire, or consistently use. If this be so, then the profession have for years been most earnestly seeking that which is entirely unnecessary and altogether superfluous, inasmuch as the *ultimatum* in this regard has long since been attained; and there is nothing remaining to stimulate professional ambition or zeal, with respect to the discovery of any new material for stopping carious teeth; and the French Academy, in offering a prize for such discovery, were manifestly behind the age.

This is equally true, of a *large* proportion of the most intelligent dentists in this country, who do not hesitate to affirm, that if a *plastic compound*, innocuous, convenient and durable, can be found, that will easily adhere to a shallow cavity, and fulfil all the intentions of gold, thousands of teeth may be annually saved, that are now sacrificed and destroyed, or removed by extraction. Dr. Cone himself is too intelligent to deny, that the proportion of dentists in the United States, who are able to pack gold foil in such a manner, in carious teeth, (especially very bad ones,) as to fulfil *every* intention, is *very small indeed*, when compared with the many who are otherwise worthy and useful practitioners. Indeed, I have never known but a very few *who claim* that they can use gold, where anything is capable of being retained, under all circumstances. But if they could, the expense of the operation is necessarily so great, (ranging from five to thirty dollars,) as utterly to debar the great mass of the community from the lasting benefits of this most valuable operation. So that those who do receive them, must be not only willing to submit to the operation, but able to incur the enormous expense. And hence, instead of the "greatest good to the greatest number," the real benefits of our noble profession would be most meanly and painfully restricted to the favoured few.

Again, what dentist need be informed that there are thousands of timid patients, so nervous and sensitive, that they cannot, nor will they submit, under *any circumstances*, to have those painful operations performed, which the experienced operator might deem advisable in their case? And now, I ask, what is duty in such a case? Shall we peremptorily dismiss our patients, or pursue the only course which is left us, viz. a *palliative treatment*? Humanity, at least, would dictate the latter.

It is just such cases as these, that have so painfully and so long divided our profession, with regard to the use of *amalgams*. And the position of each has thrown the other into an extreme, until it seems doubtful whether they ever meet again. And of this I shall utterly despair, unless, with our "*new compound*," I can bring them both together, and *cement* them in one *happy bond of union*. Dr. Keep of Boston, Drs. Baker, Lovejoy and others of New York, together with many other excellent operators have contended for amalgam, on the ground that it may be useful in those cases where *nothing* else can be used, and that duty to their patients made it necessary for them to use it.

On the other hand, it has been claimed that *amalgams* were particularly injurious to health, when placed into the cavity of a tooth, and ought *never*, under *any* circumstances, to be used for such a purpose; and that duty to the public made it necessary for them to oppose and denounce it.

I step between the two, and say, gentlemen, please to receive a *harmless* material, exceedingly convenient and easy of application, and *thus far* highly satisfactory in its results. For, while it can do *no possible* harm, (except when misapplied, and gold is liable to be thus abused,) it may do an exceeding amount of good, in mitigating human suffering, and saving time and expense.

Here, then, is a platform on which both may stand, if both will make slight concessions for the sake of union and the general good.

These considerations, it seems to me, are fully sufficient to cover the ground of objection, as presented in the very able and exceedingly interesting report above referred to, and due to the article which has fallen under the strictures of so able a pen. Other considerations favorable to its use might be urged with much propriety, but for a brief synopsis of them, I must again beg leave to refer the reader to our circular, issued in the Dental News Letter, about the first of April last. Those statements have never been gainsayed by any one, nor is it believed that they ever can be. They were carefully made, respectfully announced, and time has most triumphantly vindicated them.

